# miR-486 Responds to Apoptosis and Autophagy by Repressing SRSF3 Expression in Ovarian Granulosa Cells of Dairy Goats

**DOI:** 10.3390/ijms24108751

**Published:** 2023-05-15

**Authors:** Shujuan Liu, Qiqi Bu, Jiashun Tong, Zhanhang Wang, Jiuzeng Cui, Heran Cao, Haidong Ma, Binyun Cao, Xiaopeng An, Yuxuan Song

**Affiliations:** 1 College of Animal Science and Technology, Northwest A&F University, Yangling, Xianyang 712100, China; 2 School of Biological Science and Engineering, Shaanxi University of Technology, Hanzhong 723001, China

**Keywords:** miR-486, SRSF3, granulosa cell, apoptosis, autophagy, goat

## Abstract

The accumulation of ovarian granulosa cell (GC) apoptosis underlies follicular atresia. By comparing the previous sequencing results, miR-486 was found to be differentially expressed at higher levels in the monotocous goat than in the polytocous goat. Unfortunately, the miRNA-mediated mechanisms by which the GC fate is regulated are unknown in Guanzhong dairy goats. Therefore, we investigated miR-486 expression in small and large follicles, as well as its impact on normal GC survival, apoptosis and autophagy in vitro. Here, we identified and characterized miR-486 interaction with Ser/Arg-rich splicing factor 3 (SRSF3) using luciferase reporter analysis, detecting its role in GC survival, apoptosis and autophagy regulation through qRT-PCR, Western blot, CCK-8, EdU, flow cytometry, mitochondrial membrane potential and monodansylcadaverine, etc. Our findings revealed prominent effects of miR-486 in the regulation of GC survival, apoptosis and autophagy by targeting *SRSF3*, which might explain the high differential expression of miR-486 in the ovaries of monotocous dairy goats. In summary, this study aimed to reveal the underlying molecular mechanism of miR-486 regulation on GC function and its effect on ovarian follicle atresia in dairy goats, as well as the functional interpretation of the downstream target gene *SRSF3*.

## 1. Introduction

In mammals, female fertility potential depends on the regular growth, development and ovulation of oocytes in the ovary. Nevertheless, a significant proportion of antral follicles undergo follicular atresia during reproductive development in females [1]. Ovarian granulosa cell (GC) death is a common cause of follicular atresia [2]. On the one hand, follicular atresia has traditionally been considered to be triggered by extensive GC apoptosis, resulting in karyopyknosis and DNA fragmentation without affecting the ultrastructure of organelles [3,4]. On the other hand, autophagy also participates in follicle atresia [3]. Some apoptosis-related proteins, including p53, BCL-2, BAX and Caspase-3, respond to apoptotic signals and activate the intracellular mitochondria apoptosis pathway, while others lead to rapid cell death [5]. Nevertheless, autophagy-regulated proteins like LC3II, SQSTM1, BECN1 and ATG family members are crucial in biological processes involving the formation and degradation of autophagosomes and autolysosomes that regulate autophagy [6,7,8,9]. Indeed, apoptosis and autophagy are also collectively involved in the elimination of the majority of germ cells [10]. Li Meng et al. demonstrated that preantral and antral follicular atresia occur through distinct cell death pathways in the ovary. Antral follicular degeneration is caused by extensive GC death via apoptosis, whereas preantral follicular atresia is initiated by GC autophagy [11]. However, the mechanisms of miRNA-mediated endogenous apoptosis and autophagy in ovarian GCs are yet to be elucidated. MicroRNAs (miRNAs) are a class of endogenous, short, noncoding RNA molecules. miRNAs have emerged and have been found to be extensively involved in metabolism, homeostasis and many diseases [12,13]. In this model, miRNAs are loaded into the RNA-induced silencing complex (RISC) and combined with target transcripts with the specific reverse miRNA complementary sequence to stem protein expression, either by the degradation of target transcripts or the obstruction of protein translation [14]. Recently, miRNAs have been identified as crucial factors in ovarian follicular atresia and GC apoptosis [15]. miR-486, a key regulator, is implicated in a range of tumorigenesis, multiple diseases and other normal biological processes, including cell apoptosis and cell autophagy. For instance, the inhibition of miR-486-5p enhanced cell autophagy occurs by upregulating PTEN expression in breast cancer [16]. MiR-486 in adipose stem cell-derived exosomes induced autophagy occurs by directly targeting *SMAD1* [17]. Moreover, miR-486 mediates the apoptosis in lung alveolar epithelial cells [18], leukemia cells [19] and chondrogenic cells [20] by targeting *PTEN*, *FOXO1* and *NRF1*. In addition, miR-486 downregulated the expression of p53 and BCL-2, activating the mitochondrial apoptosis pathway to induce cardiomyocyte apoptosis [21]. Subsequently, the expression of miR-486 was dramatically upregulated in the ovaries of monotocous goats compared to polytocous goats, as determined by sequencing and bioinformatics analysis [22]. Meanwhile, another study comparing miRNA expression profiles found that the expression of miR-486 was observably increased in the GCs of the diminished ovarian reserve (DOR) compared with the normal ovarian reserve (NOR), which suggested that miR-486 highly likely influenced the GC function to generate lower developmental competence of oocytes with the DOR [23]. Taken together, these findings indicate that the differential expression of miR-486 in goat ovaries may impact GC survival and apoptosis, resulting in follicular atresia or low quality of the oocyte. Hence, we hypothesize that the distinct high expression of miR-486 in the GCs was possibly considered a potential marker to identify a monotocous goat or a polytocous goat. However, the role and molecular mechanism of miR-486 in the ovarian granulosa cells of dairy goats have not been confirmed.

Bioinformatics analysis predicted that Ser/Arg-rich splicing factor 3 (SRSF3) is a potential downstream target of miR-486. *SRSF3*, a constitutive RNA-binding protein, belongs to the Ser/Arg-rich (SR) protein family, which regulates multiple gene expression programs and various biological processes [24]. SRSF3 is known as the smallest SR family member and is composed of a single RRM and a highly phosphorylated RS domain [25]. Studies have demonstrated that SRSF3 positively contributes to several post-transcriptional and translation processes, including RNA polyadenylation, pri-miRNA formation, TAP-dependent mRNA export and protein translation [26,27]. Increasing evidence suggests that SRSF3 is a crucial potential autophagy and apoptosis regulatory factor in disease and tumorigenesis. For example, SRSF3 inhibited hypoxia-induced autophagy by enhancing the stability of SQSTM1 and suppressing BECN1 and FOXO1 expression to promote tumorigenesis [28]. In contrast, *SRSF3* silencing suppressed glioblastoma proliferation and migration and induced apoptosis [29]. Additionally, SRSF3 knockdown evoked colon cancer cell apoptosis and G1 arrest caused by reducing the protein expression of BCL-2 [30], and possibly activating p53-dependent apoptotic pathways [31]. Furthermore, through alternative splicing of p53, Caspase-2 and BCL-6, SRSF3 impacted the cellular senescence of human fibroblasts, HeLa apoptosis and transformed follicular lymphoma [32,33]. Moreover, SRSF3 is closely associated with ovarian function. Increased miRNA-124 expression significantly downregulates the target gene *SRSF3*, which eventually gives rise to the aberrant splicing regulation mechanism of SRSF3 inducing the transformation of androgens and the dysfunction of ovarian GCs [34]. In addition, mouse oocytes lacking the SRSF3 function exhibit severe maternal transcriptome damage, impaired development of fertilized-competent oocytes and abnormal blastocyst formation [26]. Until now, the endogenous regulatory roles of SRSF3 that contribute to ovarian follicular atresia and the death pathways of ovarian GCs in dairy goats have remained largely unexplored.

Considering the importance of miRNAs with respect to extensive functional and ovarian regulatory effects, we hypothesized that miR-486 plays a crucial role in GC apoptosis and autophagy-mediated follicular atresia. This study aims to investigate the apoptotic and autophagy regulatory effects of miR-486 on the ovarian GCs of dairy goats to reveal its potential molecular regulatory mechanisms. More precisely, we explore the impact of miR-486 on the regulation of GC apoptosis and autophagy in vitro, the interaction between miR-486 and its potential downstream target genes, and their involvement in the BCL-2/BAX and LC3II/SQSTM1/BECN1 pathways.

## 2. Results

### 2.1. miR-486 Accelerated GC Apoptosis

**Figure 1:**
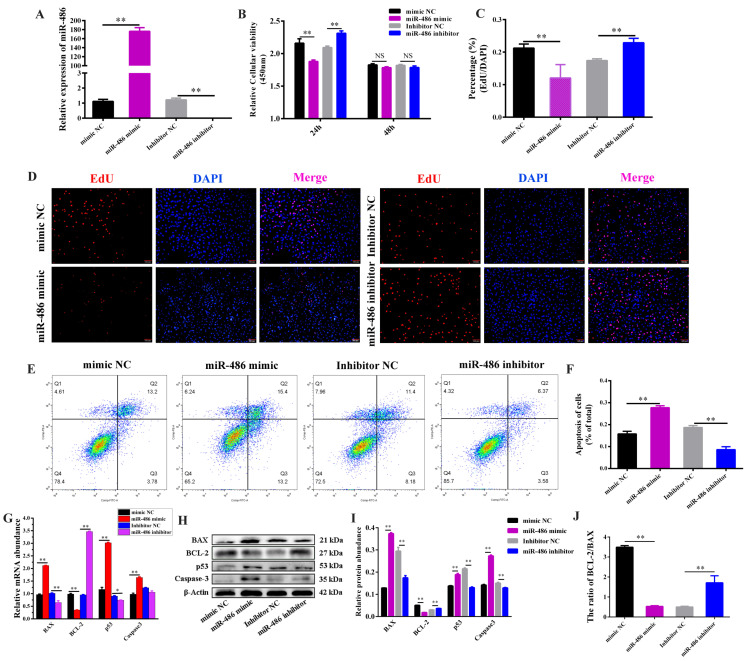
miR-486 regulated apoptosis and proliferation of granulosa cells (GCs) in dairy goats. (**A**) Transfection efficiency of the miR-486 mimic and inhibitor was detected. (**B**) GCs viability was examined using a CCK-8 assay at 24 h and 48 h after treatment with miR-486 mimic or inhibitor. (**C**,**D**) GCs were transfected with miR-486 mimic and inhibitor for 24 h. The histogram shows the proliferation percentage of GCs detected by the EdU assay. GCs in the S phase were stained red and the nuclei of the GCs were stained blue with DAPI (scale bar, 100 μm). (**E**,**F**) Flow cytometry assay. After treatment for 24 h, the percentage of apoptotic cells was calculated to evaluate the effect of miR-486 on cell apoptosis. The histogram shows the total apoptotic percentage of GCs. (**G**) mRNA expression of *BAX*, *BCL-2*, *p53* and *Caspase-3* in the GCs was assessed by RT-PCR analysis. (**H**,**I**) Cytosolic proteins were analyzed by Western blotting for BAX, BCL-2, p53 and Caspase-3 in the GCs. (**J**) The ratio of BCL-2/BAX protein expression was analyzed. Error bars represent the mean ± SEM; the mean values were considered different: * = *p* < 0.05; ** = *p* < 0.01; NS stands for not significant.

Previous sequencing results indicated that miR-486 is more highly expressed in monotocous goats than in polytocous goats [22]. To investigate the involvement of miR-486 in follicular development and atresia, we examined miR-486 expression in the diversiform tissues of Guanzhong dairy goats and the primary GCs collected from small and large follicles. Among the nine tissues in dairy goats, compared with the ovary, the mammary, kidney, liver, intestine and pituitary had lower miR-486 expression levels, while the heart, uterus and lung had higher miR-486 expression levels (Figure S1A). miR-486 was expressed at low levels in the GCs of small follicles. As the follicle developed, the expression of miR-486 was elevated in the GCs of large follicles (Figure S1B). After transfecting the miR-486 mimic and inhibitor into the GCs of small follicles, the transfection efficiency detection revealed that the miR-486 mimic significantly increased miR-486 expression and that the miR-486 inhibitor observably suppressed the intracellular expression of miR-486 (Figure 1A). Then, the function of miR-486 was verified in the GCs of small follicles. After treatment with miR-486 for 24 h, miR-486 markedly inhibited GC viability, while there was no significant effect on GC viability after transfection with miR-486 for 48 h (Figure 1B). Moreover, miR-486 notably restrained GC proliferation, as shown by EdU staining, after treatment with miR-486 for 24 h (Figure 1C,D). Flow cytometry analysis demonstrated that miR-486 transfection notably increased the total apoptosis rate of the GCs (Figure 1E,F). In addition, miR-486 positively increased *BAX*, *p53*, and *Caspase-3* mRNA and the protein levels (Figure 1G–I), but decreased BCL-2 expression and the ratio of the BCL-2/BAX protein expression (Figure 1J), suggesting that apoptosis was activated in the GCs in response to preferential miR-486 expression.

### 2.2. miR-486 Specifically Targeted SRSF3

**Figure 2:**
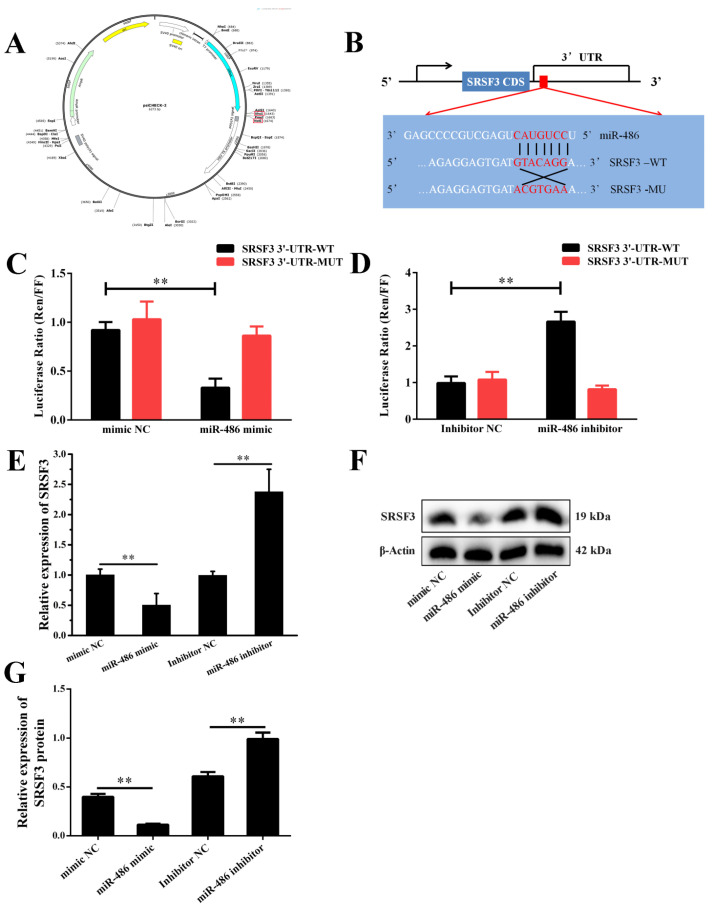
*SRSF3* is a direct target gene of miR-486 in the granulosa cells (GCs) of dairy goats. (**A**,**B**) The target site for miR-486 in the *SRSF3* 3′-untranslated region (3′-UTR) and construction of a luciferase reporter combined with the *SRSF3* 3′-UTR. WT represents the luciferase reporter vector with the *SRSF3*-WT 3′-UTR (657–663), while MU represents the luciferase reporter vector with the mutation at the miR-486 site in the *SRSF3* 3′-UTR. (**C**,**D**) After treatment with miR-486 for 24 h, the activity of double luciferase was measured. (**E**) After transfection with miR-486 for 24 h, the mRNA expression of *SRSF3* was detected by RT-PCR analysis. (**F**,**G**) The protein expression of SRSF3 was analyzed by Western blotting. Error bars represent the mean ± SEM; the mean values were considered different: ** = *p* < 0.01.

To investigate the underlying mechanisms through which miR-486 plays a prominent role in GCs, we used TargetScan (www.targetscan.org, accessed on 1 January 2023) to predict the downstream target genes of miR-486. The results revealed that the 3′UTR of *SRSF3* contained miR-486 response elements (Figure 2A,B). A luciferase reporter assay was employed to verify the binding interaction between the *SRSF3* 3′UTR and miR-486, which indicated that miR-486 dramatically decreased luciferase activity after treatment with *SRSF3*-WT in 293T cells, while it did not significantly regulate luciferase activity after joint treatment with miR-486 and *SRSF3*-MU (Figure 2C,D). Furthermore, both the mRNA and protein expression levels of *SRSF3* were obviously inhibited in the GCs after transfection with the miR-486 mimic or inhibitor, respectively (Figure 2E,F,G).

### 2.3. SRSF3 Inhibited Apoptosis of GCs

**Figure 3:**
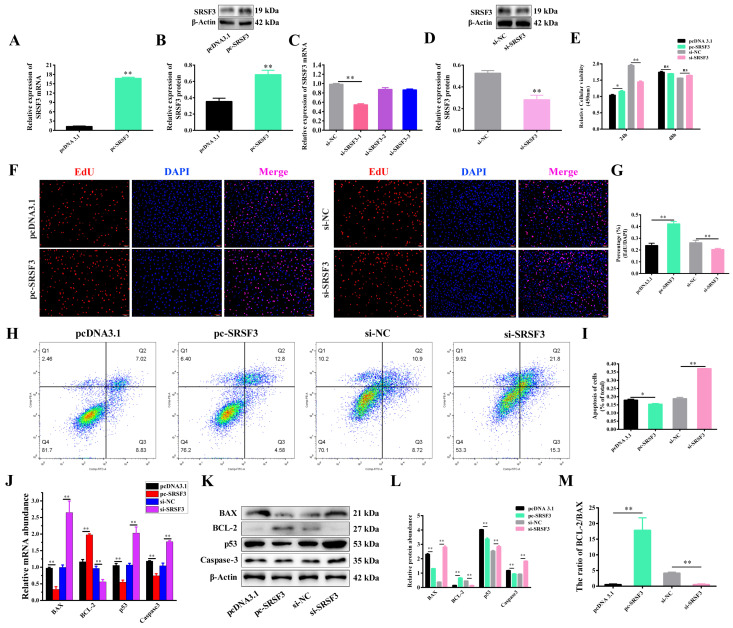
SRSF3 mediates the apoptosis and proliferation of granulosa cells (GCs). (**A**) The pc-*SRSF3* vector was successfully constructed and the overexpression of the pc-*SRSF3* mRNA was detected using RT-PCR analysis. (**B**) The protein expression of pc-*SRSF3* was analyzed. (**C**,**D**) si-*SRSF3* expression vectors were synthesized. The mRNA and protein expression of si-*SRSF3* were analyzed. (**E**) After transfection with the pc-*SRSF3* and si-*SRSF3* vector for 24 h and 48 h, cell vitality was assessed. (**F**,**G**) After treatment for 24 h, the GCs were dyed red in the S phase using the EdU assay, and the nuclei of GCs were stained blue and dyed with DAPI (scale bar, 100 μm). (**H**,**I**) The apoptotic cell percentage was analyzed using a flow cytometry assay after treatment for 24 h. (**J**) The mRNA expression of BAX, BCL-2, p53 and Caspase-3 was detected by RT-PCR analysis. (**K**,**L**) The protein expression of BAX, BCL-2, p53 and Caspase-3 and (**M**) the ratio of the BCL-2/BAX protein expression were analyzed. Error bars represent the mean ± SEM; the mean values were considered different: * = *p* < 0.05; ** = *p* < 0.01. ns stands for not significant.

SRSF3 was highly expressed in the granulosa cells (GCs) of small follicles. However, as the follicle matured, there was a notable decline in SRSF3 expression within the GCs of larger follicles (Figure S2). To further illustrate the role of SRSF3 in GC proliferation and apoptosis in small follicles, we constructed an overexpression plasmid vector (pc-*SRSF3*) and synthesized interfering RNAs (siRNAs) to overexpress and silence the expression of *SRSF3* in the GCs of small follicles. The results demonstrated that the overexpression of *SRSF3* resulted in a significant increase in *SRSF3* mRNA and protein expression in the GCs compared with the control (Figure 3A,B). Additionally, si-*SRSF3* (si-*SRSF3*-1, the most efficient of the three interfering RNAs, referred to as si-*SRSF3*) significantly inhibited *SRSF3* mRNA and protein expression in the primary GCs (Figure 3C,D). After the GCs were treated with pc-*SRSF3* for 24 h, cell viability was remarkably improved. In contrast, compared with si-NC, si-*SRSF3* observably restrained the viability of the GCs. However, no distinct difference was observed between pc-*SRSF3* and pcDNA 3.1 treatments for 24 h and 48 h (Figure 3E). Consistently, the EdU assay results showed a significant improvement in cell proliferation after 24 h of pc-*SRSF3* transfection, while the proliferation rate declined significantly after transfection with si-*SRSF3* for 24 h, indicating functional regulation through which *SRSF3* promoted GC vitality and proliferation (Figure 3F,G). Then, Annexin V and propidium iodide (PI) staining, along with flow cytometry analysis, showed that accumulated SRSF3 expression markedly reduced the late apoptosis rate (Q2) of the primary GCs (Figure 3H,I). As expected, SRSF3 negatively regulated *BAX*, *p53* and *Caspase-3* mRNA and protein levels, but increased BCL-2 expression and the ratio of the BCL-2/BAX protein expression (Figure 3J–M), suggesting that SRSF3 enhanced GC viability and proliferation while inhibiting GC apoptosis.

### 2.4. miR-486 Accelerated Apoptosis of GCs via SRSF3

**Figure 4:**
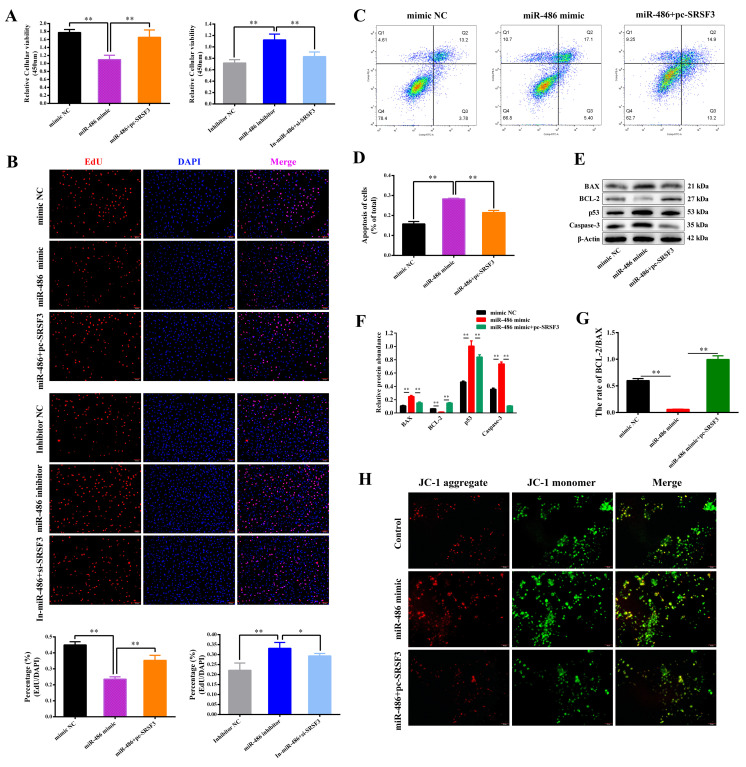
miR-486-induced apoptosis and proliferation inhibition are abolished by *SRSF3* in the granulosa cells (GCs). (**A**) After co-transfection with the miR-486 mimic and pc-*SRSF3* for 24 h, the viability of the GCs was examined via a CCK-8 kit. (**B**) The GCs were dyed red in the S phase using the EdU assay and the nuclei of the GCs were stained blue with DAPI (scale bar, 100 μm). (**C**,**D**) After treatment for 24 h, the percentage of apoptotic cells was calculated by flow cytometry. (**E**,**F**) The protein expression of BAX, BCL-2, p53 and Caspase-3 and (**G**) the ratio of BCL-2/BAX protein expression were analyzed. (**H**) The GCs were stained green and red with the JC-1 probe, and the ratio of red and green fluorescence represented the proportion of mitochondrial depolarization and the mitochondrial membrane potential level (scale bar, 50 μm). Error bars represent the mean ± SEM; the mean values were considered different: * = *p* < 0.05; ** = *p* < 0.01.

To further investigate whether SRSF3 is the downstream molecular regulatory factor of miR-486, the miR-486 mimic and pc-*SRSF3* were co-transfected into the GCs, as well as the miR-486 inhibitor and si-*SRSF3*. As detected by the CCK-8 and EdU assay, co-transfection of the miR-486 mimic and pc-*SRSF3* rescued the suppressive effect of miR-486 on GC proliferation and vitality. Co-transfection of the miR-486 inhibitor and si-*SRSF3* weakened the positive effects of the miR-486 inhibitor on the viability and proliferation of the GCs (Figure 4A,B). Co-transfection of the miR-486 mimic and pc-*SRSF3* attenuated the effect of miR-486 on the promotion of GC apoptosis, as determined by flow cytometry (Figure 4C,D). In addition, the BAX, p53 and Caspase-3 protein levels were reduced in cells co-transfected with the miR-486 mimic and pc-*SRSF3* compared with cells treated with the miR-486 mimic alone, while the protein level of BCL-2 and the ratio of BCL-2/BAX protein expression were restored after co-transfection (Figure 4E,F,G). Furthermore, co-transfection of the miR-486 mimic and pc-*SRSF3* decreased the mitochondrial membrane potential (MMP), indicating that the GCs were in the early stage of mitochondrial injury, as detected by the JC-1 probe. These results implied that the involvement of SRSF3 was able to reduce the effect of miR-486 on GC apoptosis and strengthen GC proliferation (Figure 4H).

### 2.5. miR-486 Inhibited GC Autophagy via SRSF3

**Figure 5:**
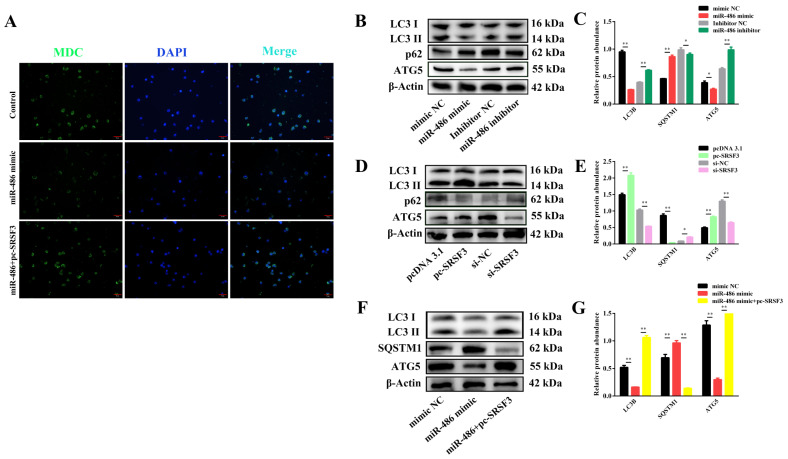
miR-486 regulated granulosa cell (GC) autophagy via SRSF3. (**A**) The autophagic vacuoles of the GCs were labeled in green using MDC staining. The nuclei of the GCs were stained blue with DAPI (scale bar, 50 μm). (**B**,**C**) After transfection with miR-486 for 24 h, the protein expression of LC3II, SQSTM1 and ATG5 was analyzed by Western blotting. (**D**,**E**) After transfection with SRSF3, the protein expression of LC3II, SQSTM1 and ATG5 was analyzed. (**F**,**G**) After co-transfection with the miR-486 mimic and pc-*SRSF3*, the protein expression of LC3II, SQSTM1 and ATG5 was analyzed. Error bars represent the mean ± SEM; the mean values were considered different: * = *p* < 0.05; ** = *p* < 0.01.

To further investigate whether miR-486 affects GC autophagy, after individual transfection of miR-486 and co-transfection of miR-486 and *SRSF3*, MDC staining of the autophagic vacuoles was used to detect the GC autophagy levels through fluorescence intensity. As shown in Figure 5A, miR-486 significantly inhibited the formation of the autophagic vacuoles compared to the control group, but the involvement of SRSF3 was able to moderate the inhibitory effect of miR-486 in the GCs (Figure 5A). The Western blot results demonstrated that the transfection of miR-486 led to decreased LC3II and ATG5 protein abundance and a remarkable increase in SQSTM1 protein levels in the GCs (Figure 5B,C). Meanwhile, *SRSF3* transfection positively promoted the accumulation of LC3II and ATG5 protein expression and the degradation of the SQSTM1 protein. However, treatment with si-*SRSF3* achieved the opposite regulatory effect (Figure 5D,E). Furthermore, to determine whether miR-486 regulates GC autophagy by targeting *SRSF3*, autophagy-related protein expression was detected in the GCs. The co-transfection results showed that compared with transfection of the independent miR-486 mimic, the degradation of LC3II and ATG5 protein abundance was rescued by SRSF3 supplementation and the SQSTM1 protein levels were observably suppressed (Figure 5F,G). These findings indicated that GC autophagy was inhibited by miR-486 treatment, while the inhibitory effect of miR-486 on autophagy could be restored with the involvement of SRSF3.

## 3. Discussion

GCs are one of most important somatic cells in the ovary [35,36]. GCs play a key role in oocyte maturation and folliculogenesis through oocyte–granulosa cell interactions. Estradiol is produced by the ovaries. During folliculogenesis, the single-layer GCs around an oocyte in the primordial follicle develop into a large number of multiple-layer GCs around an oocyte in the dominant follicle [37]. Increasing apoptosis of the GCs in mature follicles (large follicles) was a marker of poor oocyte quality and even led to limited fertilization and lambing rates [38]. The ovulation rate was the most important determinant of litter size in goats. Follicle atresia was not conducive to ovarian ovulation and even affected the litter size [39]. In this study, we assessed the moderating effects of miR-486 and the potential downstream target genes associated with the enhancement of apoptosis and the impact on autophagy of the GCs during follicular atresia to reveal a molecular regulatory mechanism through which miR-486 promotes GC apoptosis and induces follicle atresia, which results in arrested oocyte maturation and ovulation and even decreases the litter size in goats.

In this study, we found that the expression of miR-486 was lower in the GCs of small follicles (0.5–3 mm) and higher in the GCs of large follicles (3–6 mm) of Guanzhong dairy goats, which is consistent with previous transcriptome sequencing [22]. The accumulating evidence has demonstrated that the survival of both mature and atretic follicles depend on GC survival [40]. We also observed an increase in the apoptosis index with miR-486 treatment, in addition to a decrease in the proliferation of GCs in growing follicles, which might suggest that miR-486 plays an important role in follicular atresia with the development and maturation of follicles. Although intracellular metabolic processes and biological signal activation are determinants of GC survival, the understanding of miRNA-induced regulation giving rise to follicular atresia by GC apoptosis and autophagy remains incomplete. Numerous studies have indicated that miRNA-mediated regulation is now extensively valued in follicle atresia and development studies in humans, mice, cows, sows, sheep and goats [41]. Let-7 g [42], miR-10b [43], miR-15a [44], miR-23a [45], miR-26b [46] and even more differentially expressed targets during follicular atresia regulating their downstream potential target genes affected follicle atresia progression and oocyte maturation by adjusting GC proliferation, apoptosis and autophagy [4]. Our study also demonstrated a similar regulatory effect of miR-486 on ovarian GCs in Guanzhong dairy goats.

Concerning the performance of miR-486, it was as early as 2011 when Oh et al. reported that in the suppression of several pro-oncogenic traits, the inhibition of miR-486 further aggravated the proliferation of gastric cancer cells and promoted tumor progression by targeting *OLFM4* [47]. The role of miR-486 in promoting apoptosis has been widely debated in previous studies. On the one hand, mechanistic investigation has demonstrated that miR-486 induces the apoptosis of leukemia cells [19], cardiomyocytes [21] and human hypertrophic scar fibroblasts [48]. On the other hand, the results showed that miR-486-5p inhibits apoptosis of the nucleus pulposus cells in intervertebral disc degeneration, ovarian cancer cells [49], multiple myeloma cells [50] and so on. Furthermore, we found that miR-486 facilitated the apoptosis of ovarian GCs in the growing follicles of dairy goats and suppressed GC proliferation and autophagy via targeted inhibition of *SRSF3* expression. This insight will be further used to investigate the underlying molecular mechanisms of follicular atresia through which miR-486 plays a role in miR-486/SRSF3-mediated GC apoptosis and autophagy. The inhibitory effect of miR-486 on cell proliferation is already established. The property of miR-486 in inhibiting cell proliferation is evident in leukemia cells [19], acute lymphoblastic leukemia cells [51], renal cell carcinoma [52] and hepatocellular carcinoma [53], among others, corroborating our findings. Although miR-124 was an important regulator that influences the accumulation of androgens in the ovary by targeting *SRSF3*, miR-124 was not found in the differential expressed miRNAs in the sequencing results of ovaries between the monotocous goat and the polytocous goat [22]. It is possible that miR-124 is only a key regulator of the androgen receptor and not of GC survival and follicular atresia. Consequently, the regulatory role of miR-124 in influencing granulosa cell survival by targeting *SRSF3* was discounted. GC apoptosis and autophagy mainly prevail in cellular oxidative stress generating follicular atresia [4], as manifested by the changes in the mitochondrial membrane potential, the accumulation of expression of proapoptotic factors [54], changes in number of autophagosomes and partly on account of the decline in the antioxidant glutathione (GSH) [40]. Hence, the level of mitochondrial oxidative stress is critical for GC survival. In this study, we detected a mechanism involved in miR-486-induced downregulation of the mitochondrial membrane potential (MMP) responsible for generating permeabilization of the mitochondrial membrane and further promoting the release of the proapoptotic factor BAX to activate the mitochondrial apoptosis approach, and the activation pathway was inhibited by *SRSF3* overexpression. Conversely, overexpression of *SRSF3* restored the inhibitory effect of miR-486 on GC autophagy by enhancing the accumulation of LC3II and ATG5 and restraining SQSTM1 expression.

Actually, some splicing factors not only function as agents for the alternative splicing of multiple genes and mRNA stabilization, but are also involved in various post-transcriptional gene regulating processes together with noncoding RNAs (ncRNAs). MiR-9820-5p directly upregulates SRSF1 expression by binding competitively to circSLC41A1, thereby inhibiting porcine GC apoptosis [55]. CircMEF2D restrained the proliferation and differentiation of bovine myoblasts through the regulatory circMEF2D/miR-486/PI3K-AKT axis [56]. LncRNA ZNF561-AS1 enhanced colorectal cancer survival and suppressed cell apoptosis through the miR-26a-3p/miR-128-5p/SRSF6 axis [57]. Increasingly, studies have discovered that SRSF3 is broadly closely associated with cell proliferation, metastasis, apoptosis, senescence and autophagy [21]. Our results demonstrate that SRSF3 markedly fosters GC survival and inhibits GC apoptosis in dairy goats, which highlighted the distinctive antiapoptotic effect of SRSF3 on non-tumor tissue. In addition, our study demonstrated that *SRSF3* regained miR-486-mediated inhibition of autophagosome accumulation in GCs. Meanwhile, we noticed an increase in the apoptotic index caused by miR-486 through inhibition of *SRSF3* expression. Although the involvement of miR-486 in GCs gradually emerges from the apoptosis process, to maintain intracellular metabolic balance and homeostasis, autophagosomes accumulate in the GCs, combine with lysosomes and ultimately progress to degradation to provide nutrients for cell survival [58,59]. Thus, we speculated that the miR-486-mediated reduction in cumulative intracellular autophagosomes could be a pivotal process in sustaining GC survival. However, the molecular mechanism of this biological process is complex and warrants further investigation. Overall, we discovered essential roles of miR-486 in the regulation of the apoptosis and autophagy of GCs on the basis of the detected apoptosis–autophagy regulatory mechanism potential of miR-486. miR-486, highly expressed in large follicles of GCs, was able to act as a component of follicular atresia checkpoints and observably suppressed the survival of the GCs by coordinating apoptosis and autophagy through targeting *SRSF3*.

## 4. Materials and Methods

### 4.1. Primary GC Collection

Three-year-old healthy Guanzhong dairy goats in the nonestrous period with a similar age and conditions were chosen to obtain their ovaries at a local slaughterhouse. The collected ovaries were placed in PBS supplemented with streptomycin and penicillin (100 U/mL) and preserved at room temperature. After washing the ovaries with 75% alcohol and cleaning with PBS, the GCs of small follicles (0.5–3 mm) and large follicles (3–6 mm) in diameter were punctured with syringes to acquire the follicular fluid mixture. The liquid supernatant extracted from the subsided, mixed follicular fluid was centrifuged at 1500 rpm to obtain the GCs. The number of living GCs was counted using the trypan blue dye. The viable GCs (1 × 10^6^) were seeded into 6-well culture plates supported by a nutrient solution composed of the DMEM/F12 medium (Gibco, Grand Island, NY, USA), 10% fetal bovine serum (FBS), penicillin and streptomycin (100 U/mL) (Solarbio, Beijing, China), FSH (15 ng/mL) and androstenedione (5 ng/mL) (Bioniche Animal Health Inc., Belleville, Canada). The viable GCs (5.0 × 10^5^) were seeded into 12-well culture plates. The GCs were placed in an incubator at 37 °C in 5% CO_2_. In our study, the unbroken follicles were removed from the fresh ovaries. The follicles between 0.5–3 mm in diameter were collected (small follicles). The follicles between 3–6 nm in diameter were collected (large follicles). The GCs were severally acquired from small follicles and large follicles for subsequent experiments.

### 4.2. Transfection of GCs

The goat *SRSF3* CDS (XM_005696272.3) was cloned into the pcDNA3.1 vector (Thermo Fisher Scientific, Shanghai, China) between the BamH Ⅰ and EcoR Ⅰ sites, and the sequence was confirmed for accuracy. The *SRSF3* CDS forward primer: CAGGATCCCGAAATGCATCGTGATTCCTGTCCA. The *SRSF3* CDS reverse primer: CGGAATTCCGCTATTTCCTTTCATTTGACCTAGATCGGCTAC. The complete sequence of miR-486 was 5′UCCUGUACUGAGCUGCCCCGA3′. Then, the miR-486 mimic, negative control (NC), inhibitor, inhibitor NC and interfering RNA of *SRSF3* were synthesized and purchased from RiboBio Company (Guangzhou, China). When the cell density reached 85% of the cell culture dish, the RNAs and plasmid vectors were transferred into the GCs with a mediating serum-free medium (OptiMEM and Lipofectamine 2000 (Invitrogen, Carlsbad, CA, USA) for 24 h and 48 h [60].

### 4.3. Luciferase Reporter Analysis

The 315 bp sequence of the goat *SRSF3* (XM_005696272.3) 3′UTR containing the predicted miR-486 binding sites was cloned into the psiCHECKTM-2 vector (TaKaRa, Beijing, China) between the Not Ⅰ and Xho Ⅰ sites, using the total RNA extracted from the GCs as a template to extend the whole segment through PCR and sequencing. *SRSF3* 3′UTR forward primer: CCCTCGAGGGAAGACCAGTTTGCAAGAGGAGTGGT. *SRSF3* 3′UTR reverse primer: ATTTGCGGCCGCTTTATAGCTGGGCAGGAGTTAAGAGGT. The HEK293T cells were treated with 0.6 mg psiCHECK ^TM^-2-SRSF3 vectors with 10 pmol miR-486 mimic, inhibitor and controls in 24-well plates for 24 h. The fluorescence intensity of the luciferase reporters was detected via the Lipofectamine™ RNAiMAX reagent as determined by the Dual-Glo luciferase assay system (Promega, Madison, WI, USA). The results were observed for three independent repeats. The activity of Firefly was standardized as a control. An activity ratio was calculated between the luciferase obtained from firefly (red) (560 nm absorbance) and renilla (green) (480 nm absorbance). Also, the ratio of the red to green light was used to mirror the binding between miR-486 and the target genes.

### 4.4. RT-PCR Analysis

After the GCs were treated for 24 h and then washed with PBS, the total RNA was extracted from the GCs by using the TRIzol reagent (Invitrogen, Carlsbad, CA, USA). The purity and concentration of the total RNA were examined via an EPOCH microplate spectrophotometer (BioTek Instruments Inc, Winooski, VT, USA). Then, the total RNA was converted into cDNA through reverse transcription by using the PrimeScript RT reagent kit with gDNA eraser (TaKaRa, Beijing, China). The volume of the RT-PCR was 20 μL, composed of 10 μL of TB GreenPremix Ex Taq II (TaKaRa, Beijing, China), 0.8 μL of the respective forward primer and reverse primer, 2 μL of cDNA and 6.4 μL of RNase free dH_2_O. The real-time quantitative primers were synthesized by Sangon Biotech (Shanghai, China) (Table 1). The RT-PCR procedure was set at 95 °C for 10 min and then 42 cycles of 94 °C for 15 s, 60 °C for 30 s, followed by 72 °C for 30 s. The whole reaction was carried out in the CFX Connect™ real-time PCR detection system (Bio-Rad, Hercules, CA, USA). U6 and β-Actin were considered for normalization to calculate the relative expression of the genes by using the 2^−ΔΔ^Ct method. These results were analyzed for three independent repeats.

### 4.5. Western Blot Analysis

The total protein was extracted from the GCs in 6-well plates using a mixture of 1 mM PMSF and RIPA lysis buffer (Solarbio, Beijing, China) on ice. The concentration of the extracted protein was examined using the BCA assay (Sangon Biotech, Shanghai, China). Each sample of 15 μg/μL protein was separated using 12% SDS-PAGE gels. Subsequently, the proteins were transferred from the gels to PVDF membranes (Merck Millipore, Beijing, China) at 80 volts for 2 h and 20 min using a wet/tank blotting system (Bio-Rad Biotechnology, Hercules, CA, USA). Then, these membranes were blocked in TBST-configured 5% skim milk for 1 h and incubated with various primary antibodies overnight at 4 °C (Table 2), followed by full infiltration with HRP-conjugated secondary anti-mouse and anti-rabbit antibodies for 2 h. β-Actin was used for normalization to calculate the relative expression of the proteins. The proteins were visualized by using an ECL light system, and the relative expression of the protein was determined by calculating the gray value.

### 4.6. Cell Proliferation Analysis

The GCs were treated by adding 10 μL of CCK-8 solution for 3 h at 37 °C via the CCK-8 assay (ZETATM Life, Menlo Park, CA, USA). Then, the viability of the GCs was determined by the absorbance at 450 nm. The GCs were likewise seeded in 96-well plates to detect the proliferation percentage via the EdU assay (RiboBio, Shanghai, China). The GCs were treated with 50 μM EdU solution for 2 h and DAPI for 20 min at 37 °C. The detailed steps are provided in the instruction manual. The ratio of the number of GCs in the fusion diagram to the total number of GCs represented the proliferation rate of the GCs, which was verified by fluorescence microscopy. These results were analyzed for three independent repeats.

### 4.7. Cell Apoptosis Analysis

The GCs (4 × 10^5^) were cultured for 24 h and collected. The apoptosis of the GCs was detected via annexin V-FITC and propidium iodide (PI) staining using the TransDetect^®^ annexin V-FITC/PI cell apoptosis detection kit (Transgen, Biotech, Shanghai, China). The early apoptotic cells, late apoptotic cells and necrotic cells were separated and calculated to determine the apoptotic percentage using the flow cytometry method (FCM). The early stage of apoptosis was analyzed using the mitochondrial membrane potential in the GCs via a mitochondrial membrane potential assay kit with JC-1 (Beyotime, Shanghai, China). As a monomer, JC-1 emits green fluorescence, while JC-1 forms J-aggregates emitting red fluorescence. The ratio of mitochondrial depolarization was measured using the relative ratio of red to green fluorescence. These results were analyzed for three independent repeats.

### 4.8. Monodansylcadaverine (MDC) Detection

The autophagosomes of the GCs were labeled to supervise the GC autophagy vacuoles via an MDC detection kit (Solarbio, Shanghai, China), following the instructions for particular steps. The GCs were treated with 10 μL MDC (50 μmol/μL) in PBS and placed in an incubator (37 °C) for staining for 30 min. Then, the GCs were washed three times with PBS. Finally, the positive acidic vacuoles in the GCs were stained green in the perinuclear region. Images were collected with a fluorescence microscope.

### 4.9. Statistical Analysis

In this study, the data were handled with statistical software SPSS 22.0 (IBM, New York, NY, USA) and analyzed using Student’s *t* test or analysis of variance, according to the mean ± standard deviation (SD). Statistically, the differences were representative with *p* < 0.05. Generally, these results were analyzed for three independent repeats.

## 5. Conclusions

miR-486, which is highly expressed in large follicles, promoted GC apoptosis in Guanzhong dairy goats by targeting *SRSF3* and inhibited GC proliferation and autophagy. SRSF3, which was expressed at low levels in large follicles, markedly promoted GC vitality and proliferation. Our study clarified a potential regulatory mechanism through which miR-486 induced GC apoptosis and inhibited GC autophagy by inhibiting SRSF3 expression, which suggests that miR-486 might represent a potential target for follicular atresia in dairy goats.

## Figures and Tables

**Table 1:** Real-time quantitative primers.

Gene Name	Primer	Primer Sequences (5′-3′)	Product Size (bp)
miR-486	RT primer	GTCGTATCCAGTGCAGGGTCCGAGGTATTCGCACTGGATACGACTCGGGG	
	FORWARD	GCGCGTCCTGTACTGAGCTG	
	REVERSE	AGTGCAGGGTCCGAGGTATT	
Negative control (NC)	FORWARD	UUCUCCGAACGUGUCACGUTT	
	REVERSE	ACGUGACACGUUCGGAGAATT	
U6	FORWARD	GTGCTCGCTTCGGCA GCACAT	
	REVERSE	ATCCAGTGCAGGGTCCGAGG	
BCL2	FORWARD	AGAGCGTCAACCGGGAGATG	167
	REVERSE	CAGCCAGGAGAAATCAAACAGG	
BAX	FORWARD	CATCAACTGCCTTGGACTTT	130
	REVERSE	GACCACTCCTCCCTACCCT	
Caspase-3	FORWARD	ATACCAGTTGAGGCAGAC	161
	REVERSE	TTAACCCGAGTAAGAATGT	
P53	FORWARD	CCCCTTCCCTCAACAAGC	144
	REVERSE	GCCTCACAACCTCCGTCA	
β-Actin	FORWARD	CACGGTGCCCATCTACGA	158
	REVERSE	CCTTGATGTCACGGACGATTT	

**Table 2:** Primary antibodies.

Antibody	Manufacturer	Product ID
Anti-p53	BBI Life Science	D120082
Anti-CASP3	BBI Life Science	D160009
Anti-β-Actin	BBI Life Science	D190606
SRSF3	ABCAM	AB198291
BCL2	ABclonal	A16776
BAX	ABclonal	A15633
LC3II	Abways	CY5528
SQSTM1	Abways	CY5546
ATG5	Abways	CY5766

## Data Availability

Not applicable.

## Supplementary Materials

The following supporting information can be downloaded at: https://www.mdpi.com/article/10.3390/ijms24108751/s1.
