# Supplementary material for: miR-486 Responds to Apoptosis and Autophagy by Repressing SRSF3 Expression in Ovarian Granulosa Cells of Dairy Goats
Source: Int J Mol Sci. 2023 May 15;24(10):8751. doi: 10.3390/ijms24108751 (PMC10217930; doi:10.3390/ijms24108751)
Supplement: Supplementary file 1 [file ijms-24-08751-s001.zip › ijms-2315219-supplementary.pdf]

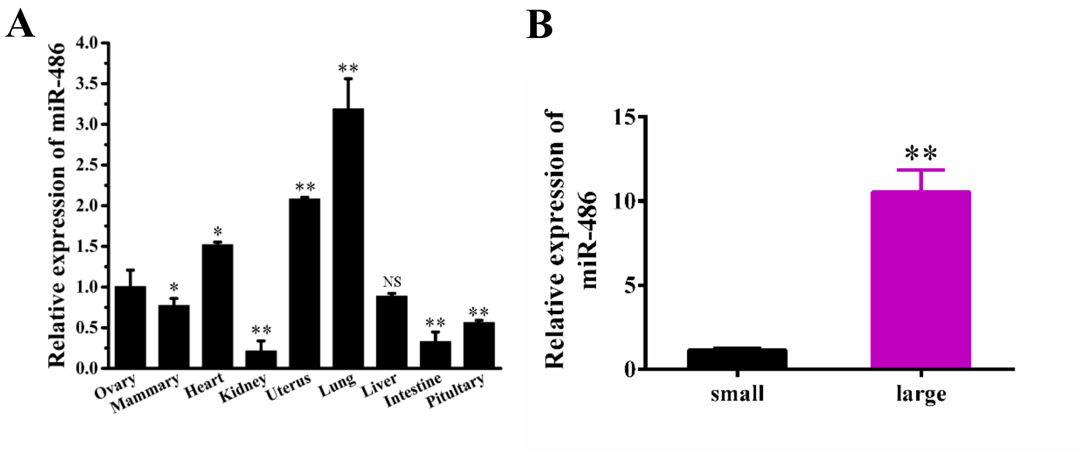

Figure S1. The mRNA expression of miR-486 in various tissues and GCs of follicles. (**A**) The mRNA expression of miR-486 was detected among ovary, mammary, kidney, heart, uterus, lung liver, intestine, pituitary. (**B**) The mRNA expression of miR-486 was analyzed in small and large follicles. * = $p < 0.05$; ** = $p < 0.01$; NS stands for Not Significant.

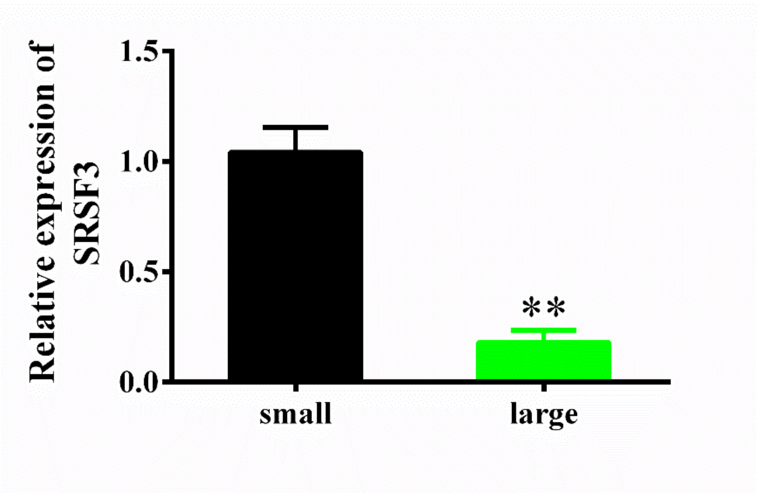

Figure S2. The mRNA expression of SRSF3 was analyzed in GCs of small and large follicles. ** = $p < 0.01$.
